# Quorum Quenching Agents: Resources for Antivirulence Therapy

**DOI:** 10.3390/md12063245

**Published:** 2014-05-30

**Authors:** Kaihao Tang, Xiao-Hua Zhang

**Affiliations:** College of Marine Life Sciences, Ocean University of China, Qingdao 266003, China; E-Mail: kaihao.tang@gmail.com

**Keywords:** quorum sensing, quorum quenching, marine, AHL-degrading activity, antivirulence therapy, antibiotic resistance

## Abstract

The continuing emergence of antibiotic-resistant pathogens is a concern to human health and highlights the urgent need for the development of alternative therapeutic strategies. Quorum sensing (QS) regulates virulence in many bacterial pathogens, and thus, is a promising target for antivirulence therapy which may inhibit virulence instead of cell growth and division. This means that there is little selective pressure for the evolution of resistance. Many natural quorum quenching (QQ) agents have been identified. Moreover, it has been shown that many microorganisms are capable of producing small molecular QS inhibitors and/or macromolecular QQ enzymes, which could be regarded as a strategy for bacteria to gain benefits in competitive environments. More than 30 species of marine QQ bacteria have been identified thus far, but only a few of them have been intensively studied. Recent studies indicate that an enormous number of QQ microorganisms are undiscovered in the highly diverse marine environments, and these marine microorganism-derived QQ agents may be valuable resources for antivirulence therapy.

## 1. Introduction

Antibiotics are recognized as effective antimicrobial agents for curing diseases caused by pathogenic bacteria. Traditional antibiotics are bactericidal or bacteriostatic by targeting essential processes for bacterial growth including cell wall synthesis, DNA replication, RNA transcription and protein synthesis [1]. However, because of the life-or-death selective pressure imposed on the targeted pathogens, antibiotic-resistant strains are constantly emerging. The inappropriate and excessive use of antibiotics accelerates the emergence of antibiotic resistance. Unfortunately, in contrast to the rising levels of antibiotic resistance, the pace of novel antibiotic development has severely slowed in the preceding few decades. This problem leads to the urgent need for the development of new antimicrobial agents targeting virulence (toxin function and delivery, regulation of virulence expression and bacterial adhesion) rather than the essential processes of pathogenic microorganisms [1]. The so-called antivirulence therapy may impose less selective pressure on pathogenic microorganisms, and in theory, decrease resistance [1].

Quorum sensing (QS) is a process for bacteria to communicate, regulate gene expression and synchronize social behaviors, such as biofilm formation, bioluminescence and secretion of virulence factors [2,3]. Hitherto diverse molecular mechanisms of sophisticated QS have been unraveled [2]. In particular, it has been established that QS regulates the secretion of virulence factors in many pathogens, such as * Pseudomonas aeruginosa*, *Erwinia carotovora*, *Vibrio* spp. and *Burkholderia* spp. [2]. In addition, these pathogens use QS to regulate biofilm formation, which is a critical defense against antibacterial drugs or the immune system of a host. Because QS is not essential for the growth of bacteria, quenching QS (quorum quenching, QQ) in these pathogens would disarm virulence rather than kill the bacteria, which may considerably weaken the selective pressure imposed on the pathogens and delay the evolution of resistance to QQ drugs. All of these features make QS an ideal target for antivirulence therapy [1].

Here, we provide an overall summary of the uniformity and diversity of QS in microorganisms, as well as the QS circuits in some representative species. Two main QQ agents, *i.e.*, small molecular QS inhibitors and macromolecular QQ enzymes, are discussed. The current status and trends of antivirulence therapy utilizing QQ resources from the marine environment are considered. Based on this knowledge, a critical appraisal and perspective of the resource for antivirulence in the environment is presented.

## 2. The Distribution of QS Systems in Microorganisms

The term QS was first proposed to describe the phenomenon that marine bacteria, *i.e.*, *V. fischeri* and *V. harveyi*, use signaling molecules (autoinducers, AIs) as sensors of cell density so that the population as a whole may coordinate the social behavior of bioluminescence [4]. However, in addition to cell density, the concentration of AIs in the natural environment is determined by many other biotic and abiotic factors, such as the spatial distribution of cells and the diffusional characteristics of the environment [5,6,7,8,9]. Redfield [5] proposed a concept of diffusion sensing (DS) to challenge the previously assumed role of QS. She argued that cells employ AIs to assess diffusive properties of the environment, and thereby determine when to produce more costly secreted molecules, such as extracellular protease. This may be less effective in the environment with a high diffusion rate. Another concept of efficiency sensing (ES) was introduced in an attempt to unify both QS and DS [6]. In particular, it has been demonstrated that even a single cell may initiate QS-regulated behaviors by physical confinement [10,11], which provides support for the hypotheses of DS and ES. However, QS and DS are not diametrically opposed, and the utility of ES has been debated [8,12]. In addition, many other hypotheses emphasizing different factors have been suggested to compete or unite with QS [13]. However, Platt *et al.* [13] argued that the introduction of new hypotheses would lead to confusion rather than clarification. Therefore, it was suggested that the processes of QS could be viewed broadly with full awareness of the effects of environmental factors [13].

Despite the presence of distinct QS systems in different microorganisms, the fundamental processes are similar. Initially, AIs are synthesized by AI synthases and diffuse away. When the concentrations of AIs increase to a threshold, AIs are detected by receptors. Subsequently, the AI-bound receptors activate the expression of relevant genes, including AI synthase-encoding genes. This results in a positive feed-back loop for biosynthesis of AIs, which may be able to promote the synchrony among a population.

**Figure 1:**
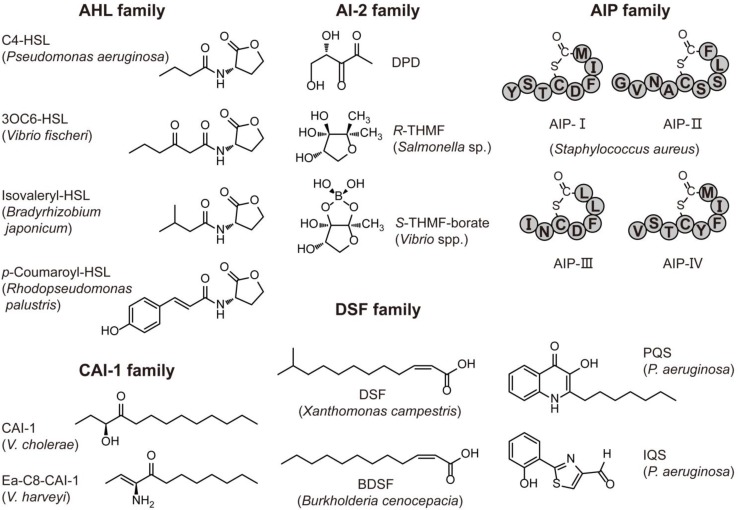
Structures of representative quorum sensing (QS) signals.

AHL: *N*-Acyl-homoserine lactonse; AI-2: Autoinducter-2; AIP: Autoinducing peptides; CAI-1: *Cholerae* autoinducer-1; Ea-C8-CAI-1: (*Z*)-3-Aminoundec-2-en-4-one; DSF: Diffusible signal factor; BDSF: *Burkholderia cenocepacia* diffusible signal factor; PQS: *Pseudomonas* quinolone signal; IQS: Integrating QS signal; *R*-THMF: (*2R*,*4S*)-2-Methyl-2,3,3,4-tetrahydroxytetrahydrofuran ; *S*-THMF-borate: (*2S*,*4S*)-2-Methyl-2,3,3,4-tetrahydroxytetrahydrofuran-borate; DPD: 4,5-Dihydroxy-2,3-pentanedione.

Increasing evidence has revealed that QS is prevalent in bacteria, fungi and archaea (Figure 1, Table 1). For example, it is widely accepted that *N*-acylhomoserine lactones (AHLs) and autoinducing peptides (AIPs) are mainly used by Gram-negative and Gram-positive bacteria for intraspecies communication, respectively. Autoinducter-2 (AI-2) signals are hypothesized to be used for interspecies communication because AI-2 production and the synthase LuxS homologues are widespread among Gram-negative and Gram-positive bacteria [14,15]. However, many bacteria only produce AI-2, but lack cognate receptors. The two classes of AI-2 receptors, LuxPQ and Lsr-receptor, are restricted to Vibrionales representatives and pathogenic bacteria associated with endotherms, respectively [16,17]. Therefore, the QS role of LuxS protein is arguable. It may be only a metabolic enzyme involved in the activated methyl cycle (AMC) [18] in these bacteria that are devoid of a complete AI-2 signaling pathway [16]. In addition to AI-2, indole has been suggested as an interspecies signal molecule, because it is shared by 85 species of Gram-positive and Gram-negative bacteria [19] (Table 1). However, some exceptions have been discovered. One type of AHL, 3-oxo-octanoyl homoserine lactone (3OC8-HSL), is utilized by a Gram-positive bacterium, *Exiguobacterium* sp. MPO, as a QS signaling molecule to regulate biofilm formation and extracellular polymeric substance production [20]. In addition, peptide-based QS is also found in a hyperthermophilic Gram-negative bacterium, *Thermotoga maritima* [21], and additionally in the yeast *Cryptococcus neoformans* [22]. Furthermore, AHLs are not the only signal type employed by Gram-negative bacteria. The diffusible signal factor (DSF) family, *V. cholerae* autoinducer-1 (CAI-1) family and other particular signals, such as *Pseudomonas* quinolone signal (PQS), integrating QS signal (IQS) and pyrone signal are also employed by some Gram-negative bacteria (Table 1). Among these molecules, the CAI-1 family is found mainly in *Vibrio* spp. [23] whereas the DSF family commonly exists in some plant pathogens, such as *Xanthomonas* and *Burkholderia* spp. [24]. Likewise, social behaviors namely filamentation and biofilm formation by the opportunistic fungal pathogen *Candida albicans* are regulated by farnesol- and tyrosol-based QS [25,26]. Moreover, it is striking that AHLs are present in more microorganisms than originally expected. Recently, a novel type of AHL, *N*-carboxyl-acyl-homoserine lactones, was found in a methanogenic archaeum *Methanosaeta harundinacea* to regulate its filamentous growth [27]. Each of these signal families has different structures and is used by different microbial groups (Figure 1, Table 1). These diverse signals may allow microbial populations to differentiate themselves from others, so as to synchronize and coordinate social behaviors.

**Table 1:** Quorum sensing (QS) systems of microorganisms.

QS Signaling Type	Structure	Representative Microorganisms	Associated Phenomena	Reference
**Intraspecies Communication Signals**				
“Traditional” AHL	C4-C18, 3OC4-3OC18 and 3OHC4-3OHC18	Various Gram-negative bacteria; only one Gram-positive bacteria: *Exiguobacterium* sp. MPO	Virulence, biofilm, swarming and bioluminescence	[2,20]
“Noncanonical” AHL	*p*-Coumaroyl-HSL	*Rhodopseudomonas palustris* CGA009	Global gene expression	[28]
	Cinnamoyl-HSL	*Bradyrhizobium* spp.	Not identified	[29]
	Isovaleryl-HSL	*B. japonicum* USDA110	Not identified	[30]
	*N*-Carboxyl-acyl-HSL	Archaeum *Methanothrix harundinacea*	Filamentous growth	[27]
DSF family	*Cis*-unsaturated fatty acid	*Xanthomonas* spp., and *Burkholderia cenocepacia*	Virulence, biofilm and antibiotic tolerance	[24]
CAI-1 family	α-Hydroxyketones	*Vibrio* spp. and *Legionella pneumophila*	Virulence and biofilm	[23,31]
AIP family	Linear or cyclized oligopeptide	Many Gram-positive bacteria; only one Gram-negative bacterium: *Thermotoga maritima*	Virulence, biofilm, sporulation and exopolysaccharide production	[21,32]
PQS or IQS	Quinolone or thiazole compounds	*Pseudomonas aeruginosa*	Virulence and biofilm formation	[33,34]
Pyrones	α-Pyrones	*Photorhabdus luminescens*	Virulence	[35]
**Interspecies and Interkingdom Communication Signals**				
AI-2	*S*-THMF-borate *R*-THMF	Many Gram-negative and Gram-positives bacteria	Virulence and biofilm formation	[15]
AI-3	Unknown	Enterohemorrhagic *Escherichia coli* (EHEC)	Virulence	[36]
Indole	2,3-Benzopyrrole	Many Gram-negative and Gram-positives bacteria	Virulence and biofilm formation	[19]
**QS Signals in Fungi**				
Farnesol or Tyrosol	Sesquiterpene or phenylethanoid	*Candida albicans*	Inhibition or stimulation filamentation and biofilm formation	[25,26]
Peptide	NH_2_-NFGAPGGAYPW-COOH	*Cryptococcus neoforman*s	Colony formation in agar media	[22]

AHL: *N*-Acyl-homoserine lactonse; AI-2: Autoinducter-2; AI-3: Autoinducter-3; AIP: Autoinducing peptides; DSF: Diffusible signal factor; CAI-1: *Cholerae* autoinducer-1; PQS: *Pseudomonas* quinolone signal; IQS: Integrating QS signal.

In most cases, one species usually employs a single type of QS. However, some species may harbor multiple QS systems. For example, *V. harveyi* utilizes a four-channel integrated QS system (AHL-, CAI-1-, AI-2- and nitric oxide-dependent channels) [37,38], whereas *V. fischeri* possesses a multichannel system (C8-HSL- and AI-2-dependent channels) and an additionally LuxI/R circuit (3OC6-HSL) [37]. *P. aeruginosa* employs various QS systems, including two complete AHL-dependent QS circuits (RhlI/R and LasI/R circuits), a 3OC12-HSL-responsive orphan receptor QscR, a *Pseudomonas* quinolone signal (PQS)-dependent QS, and a recently identified IQS (integrating the QS network) signal, which together compose a hierarchical QS network to regulate virulence expression and respond to environmental stress [33,39].

### 2.1. Characteristics of AHL Molecules

AHL-dependent QS exists in many pathogenic bacteria. A typical AHL molecule consists of a homoserine lactone and an acyl chain with an even number of carbons (C4-C18) together with an occasional modification at the C3 position (hydroxy or olefinic double bond) [2]. The shortest and longest AHLs found in nature are C4-HSL and C18-HSL, respectively. Recently, some special AHLs with novel structures have been discovered in Gram-negative bacteria and archaea, and include aryl-homoserine lactone (*p*-coumaroyl-HSL and cinnamoyl-HSL) [28,29], branched-chain fatty acyl-homoserine lactone (isovaleryl-HSL) [30] and *N*-carboxyl-acyl-homoserine lactone [27] (Table 1).

The solubility, diffusibility and stabilization of AHLs are correlated with their structures. Generally, the solubility and diffusibility will be increased, and the stabilization decreased along with the shorter length of acyl chain of AHLs. For example, 3OC6-HSL used by *V. fischeri* shows higher solubility and diffusibility which enables diffusion inside and outside of cells [2]. Conversely, 3OC12-HSL in *P. aeruginosa* can only be transported through efflux pumps [2]. In addition, the modification at the C3 position (also termed the β position) may increase the solubility of AHLs. AHLs are susceptible to pH and temperature of the surrounding environment. In fact, alkaline pH and high temperatures will promote their abiotic hydrolysis [40]. Therefore, it seems appropriate that the hyperthermophilic Gram-negative bacterium *Thermotoga maritima* utilizes peptides as the QS signal rather than AHLs in its hydrothermal habitat [21]. Another case is that of the photosynthetic bacterium, *Rhodopseudomonas palustris*, which uses an AHL synthase to produce *p*-coumaroyl-HSL from environmental *p*-coumaric acid, which is a plant metabolite, rather than fatty acids from cellular pools. This indicates that *R. palustris* QS might have evolved to fit its surrounding [28].

### 2.2. The Diversity and Uniformity of QS Systems

**Figure 2:**
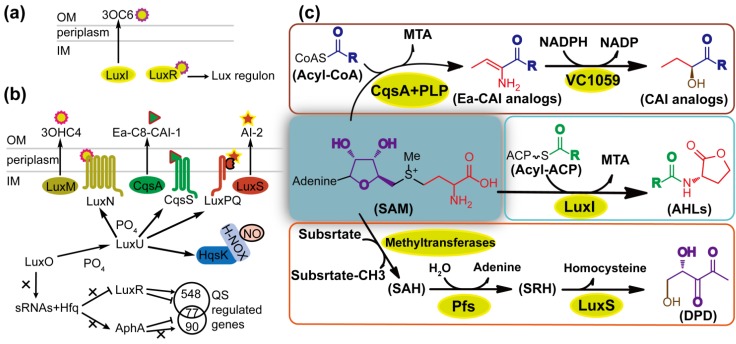
Representative QS circuits and autoinducer biosynthesis. (**a**) LuxI/R circuit of *Vibrio fischeri.* OM: outer membrane. IM: inner membrane; (**b**) QS circuit of *V. harveyi*; (**c**) Biosynthesis of *N*-acylhomoserine lactones (AHLs), *V. cholerae* autoinducer-1 (CAI-1) analogs and 4,5-dihydroxy-2,3-pentanedione (DPD). Differently colored carbons, nitrogens and oxygens show where they are derived. See details in the text.

The LuxI/R circuit in marine bioluminescent *V. fischeri* (Figure 2a) and the integrated QS of *V. harveyi* are good examples to explain how different QS circuits work in a similar way (Figure 2b). *V. harveyi* possesses a sophisticated QS system consisting of four parallel channels, *i.e.*, LuxM/N (AHL-dependent), LuxS/PQ (AI-2-dependent), CqsA/S (CAI-1-dependent) [2] and NO/H-NOX/HqsK (nitric oxide-dependent) [38]. In AHL-dependent QS, HAI-1 (3OHC4-HSL) is synthesized by the synthase LuxM. With AI-2-dependent QS, DPD (4,5-dihydroxy-2,3-pentanedione) is synthesized by LuxS, and is spontaneously cyclized and hydrated to form *R*-THMF and *S*-THMF (Figure 1). *R*-THMF may be detected by *Salmonella enterica* serovar Typhimurium directly [41], whereas, in the presence of boron, *S*-THMF is further catalyzed to form *S*-THMF-borate that can be utilized by *Vibrio* species as the AI-2 signal. In CAI-1-dependent QS, Ea-C8-CAI-1 is synthesized by CqsA [42]. The cognate receptors LuxN, LuxPQ, and CqsS are bi-functional two-component enzymes that possess both kinase and phosphatase activities [2]. LuxP is a periplasmic protein in complex with LuxQ; the former detects signals, and the latter transfers phosphate [2]. In the NO-responsive QS, NO is sensed by H-NOX and the NO/H-NOX complex regulates kinase and phosphatase activities of HqsK [38]. LuxR and AphA are the primary and secondary master regulators of QS in *V. harveyi*, respectively. At low cell density (LCD) or low NO concentration, phosphates from unliganded receptors are transduced to a single phosphotransfer protein LuxU, and subsequently to LuxO. Phospho-LuxO, together with a sigma factor σ^54^, activates the transcriptions of five small regulatory RNAs. Small RNAs repress the translation of LuxR and activate the translation of AphA [43,44]. At high cell density (HCD) or high NO concentration, phosphates are conversely transferred to the AI/NO-bound receptors and thereby the production of AphA is depressed whereas LuxR protein is maximally produced to regulate the expression of target genes [2]. Actually, LuxR is also present at LCD, whereas little or no AphA is present at HCD [45]. The two master regulators AphA and LuxR regulate 167 genes and 625 genes, respectively, and coregulate 77 genes, thereby generating a precise temporal pattern of gene expression [45].

Intriguingly, the biosynthesis pathways of AHLs, AI-2 and CAI-1 are related to each other and involved in the AMC, which is an important metabolic pathway responsible for the generation of the cell major methyl donor *S*-adenosylmethionine (SAM) and the recycling of methionine by detoxification of *S*-adenosyl-L-homocysteine (SAH) (Figure 2c). AHL is synthesized from SAM and acyl carrier protein (acyl-ACP) by LuxI-type synthase [2]. The structurally conserved homoserine lactone of AHL molecule is derived from SAM whereas the variable acyl tail is assembled from fatty acid. CAI-1 is synthesized from SAM and acyl-coenzyme A via a multistep reaction involving CsqA, pyridoxal phosphatase (PLP) and VC1059 [23]. CAI-1 and Ea-CAI-1 contain single three-carbon units, being derived from SAM and subsequently attached to an acyl tail. In AI-2 biosynthesis, the release of the activated methyl group from SAM to an acceptor molecule gives rise to a toxic intermediate, SAH, which is converted by 5′-methylthioadenosine/sadenosylhomocysteine nucleosidase (MTAN/Pfs) to *S*-ribosylhomocysteine (SRH) [46]. LuxS catalyzes the cleavage of SRH to homocysteine and DPD, which further undergoes spontaneously cyclization and hydration reactions to be mature AI-2 [46]. Unlike AHLs and CAI-1, all the carbons of DPD are derived from SAM.

All of the discovered QS circuits share three critical steps: 

(1) production of signal molecules,

(2) release of signal molecules, and

(3) recognition of signal molecules by receptors.

In most cases, the LuxR-type transcriptional regulators are able to activate target genes, whereas the EsaR in *Pantoea stewartii* functions as a transcriptional repressor. Unliganded EsaR binds DNA and represses transcription. The binding of AI to EsaR promotes DNA release and thereby promotes gene expression [47]. Also, QS in Gram-positive bacteria contains three steps. Unlike AHL-dependent QS, AIPs are secreted to the environment by specialized transporters and subjected to post-translation modification to become mature linear or cyclized AIPs during transport [32].

## 3. Natural Quorum Quenching Resources

Quorum quenching is now referred to as the interference with QS, and may reverse the QS regulated phenotypes, such as virulence. Because QS is not essential for the growth of bacteria, quenching QS has been recommended as a promising strategy for antivirulence therapy. In particular, diverse QQ agents have been identified from various sources and organisms. All of the QQ agents may be classified into two groups according to their molecular weight, *i.e.*, small molecular and macromolecular QQ agents, which are also referred to as QS inhibitors and QQ enzymes, respectively.

The words “quorum quenching” and “QS inhibitors” were first used to describe the enzymatic degradation of AHL signals [48] and inhibition of QS by small molecular antagonists [49], respectively. Here, in order to avoid a semantic quagmire, the word “quorum quenching” is used in its most extensive connotation to describe any forms of interference with QS, including QS inhibitors and QQ enzymes.

### 3.1. Small Molecular QQ Agents

There is abundant literature about the identification of novel and effective QS inhibitors from natural products and synthetic molecules. Multiple methods have been applied to identify QS inhibitors: purification from crude extracts of candidate organisms, random and high-throughput screening of QS inhibitors from compound libraries, and computer-aided screening from 3D structure libraries of known compounds. However, QS biosensors, which are genetically manufactured strains that express reporter genes (e.g., *lacZ*, *gfp* or *luxCDABEG*) in response to specific QS signals, are potent and indispensable tools for identifying QS inhibitors.

Natural QS inhibitors with highly diverse structures possess inhibitory activities against AHL, PQS, AI-2 and AIP-dependent QS (Table 2). It is notable that there are some unusual forms of interference with QS. Because the virulence factors of many pathogens are regulated positively by QS, it is reasonable to control pathogenicity by inhibiting QS. On the contrary, the orphan receptor QscR of *P. aeruginosa* [50] and CAI-1-dependent QS in *V. cholerae* [51] are negatively correlated with virulence, which means that these two particular pathways should be activated rather than inhibited for reducing virulence. Various synthetic agonists of each circuit have been identified [52,53,54]. However, natural compounds with this activity have not yet been found except for some corresponding AIs analogs, e.g., 3OC10-HSL for QscR and Ea-C8-CAI-1 for CqsS of *V. cholerae*. Therefore, specific screening work could be carried out to explore more natural QS inhibitors.

#### 3.1.1. Marine-Derived Inhibitors against AHL-Dependent QS

AHL-dependent QS inhibitors account for the largest proportion of natural QS inhibitors (Table 2). Marine-derived QS inhibitors have been found in marine bacteria, fungi, algae, bryozoan, corals and sponges.

An increasing number of studies indicate that marine cyanobacteria have become one of the richest resources for biologically active and structurally unique natural products. Many AHL-dependent QS inhibitors with divergent structures have been discovered in several species of marine cyanobacteria (Table 2). The tumonoic acids (E, F, G and H), which have been isolated from *Blennothrix cantharidosmum*, inhibit the bioluminescence of *V. harveyi* BB120 without affecting bacterial growth. Here, tumonoic acid F is the most active moiety (IC_50_ of 62 μM) [55]. Moreover, *Lyngbya majuscula* produces four different compounds, including malyngolide (MAL), 8-epi-malyngamide C, lyngbic acid and lyngbyoic acid, which inhibit LasR of *P. aeruginosa* responding to exogenous 3OC12-HSL [56]. Interestingly, honaucins A–C [57], isolated from *Leptolyngbya crossbyana*, possess dual-inhibitory activity against both QS and inflammation, which may represent a new strategy for developing multi-functional drugs. It is noteworthy that marine cyanobacteria produce many different kinds of AHL-dependent QS inhibitors, and most likely have evolved mechanisms to control the associated microbial communities by interfering with their cell-to-cell communication.

**Table 2:** Natural QS inhibitors.

Category	Species	Inhibitor	Target	Reference
**Marine-Derived Inhibitors against AHL-Dependent QS**				
Actinobacteria	*Streptomyces* sp.	Piericidin A1	CviR	[58]
Bacteria	*Halobacillus salinus*	*N*-(2-Phenylethyl)-isobutyramide and 3-Methyl-*N*-(2-phenylethyl)-butyramide	LuxR, CviR and *Vibrio harveyi*	[59,60]
Bacteria	*Bacillus cereus* D28	Cyclo-l-proline-l-tyrosine	*Chromobacterium violaceum*	[60]
Bacteria	*Marinobacter* sp. SK-3	Diketopiperazines (dkps)	CviR and LuxR	[61]
Bryozoan	*Flustra foliacea*	Brominated alkaloids compounds	CepR and LuxR	[62]
Coral	*Pseudoplexaura flagellosa* and *Eunicea knighti*	Cembranoids	LuxR and *V. harveyi*	[63,64]
Cyanobacteria	*Blennothrix cantharidosmum*	Tumonoic acid F	*V. harveyi*	[55]
Cyanobacteria	*Lyngbya majuscula*	8-*Epi*-malyngamide C and lyngbic acid	LasR	[65]
Cyanobacteria	*L. majuscula*	Lyngbyoic acid	LasR	[66]
Cyanobacteria	*L. majuscula*	Malyngolide	CviR and LasR	[56]
Cyanobacteria	*Leptolyngbya crossbyana*	Honaucins A–C	LuxR	[57]
Cyanobacteria	*Lyngbya* sp.	Pitinoic acid A	LasR	[67]
Cyanobacteria	*Lyngbya* sp.	Pepitdes (microcolins A and B)	LuxR	[68]
Fungi	*Penicillium atramentosum*	Crude extracts	LasR	[69]
Red algae	*Ahnfeltiopsis flabelliformes*	Floridoside, betonicine and isethionic acid	TraR	[70]
Sponge	*Luffareilla variabilis*	Manoalide, manoalide monoacetate, and secomanoalide	LuxR and LasR	[71]
Sponge	*Hymeniacidon aldis*	Alkaloid (hymenialdisin)	LuxR and LasR	[68]
**Terrestrial-Derived Inhibitors against AHL-Dependent QS**				
Bacteria	*Staphylococcus delphini*	Yayurea A and B	LuxN	[72]
Bacteria	*Stenotrophomonas maltophilia* BJ01	*Cis*-9-octadecenoic acid	CviR	[73]
Bacteria	*Pseudomonas* spp*.*	Protoanemonin	LasR	[74]
Fungi	*Aspergillu*s spp.	Kojic acid	LuxR	[68]
Fungi	*Penicillium* spp.	Patulin and Penilillic acid	LasR and RhlR	[75]
Insect productions	Bee	Honey and propolis	*C. violaceum*, LasR and RhlR	[76,77]
Insect: fire ant	*Solenopis invicta*	Solenopsin A	*rhl* circuit	[78]
Plant	*Baccharis cassinaefolia*	Benzopyran	CviR, LuxR and LasR	[68]
Plant	*Syzygium aromaticum*	Eugenol	CviR, LasR and PQS	[79]
Plant: alfalfa	*Medicago sativa*	l-Canavanine	CviR and ExpR	[80]
Plant: *Combretaceae*	*Combretum albiflorum*	Catachin and naringenin	CviR and RhlR	[81,82]
Plant: *Compositae*	*Centratherum punctatum*	Sesquiterpene lactones	*Pseudomonas aeruginosa*	[83]
Plant: garlic	*Allium sativum*	Ajoene	LuxR family	[84]
Plant: horseradish	*Armoracia rusticana*	Iberin	LasR and RhlR	[85]
Plant: *Myristicaceae*	*Myristica cinnamomea*	Malabaricone C	CviR	[86]
Plant: tree	*Drimys winteri*	A drimane sesquiterpene	*C. violaceum*	[87]
Plant: turmeric	*Curcuma longa*	Curcumin	CviR	[88]
Plants	Various plants	*p*-Coumaric acid	PpuR, CviR and TraR	[89]
Plants and bacteria	*Conocarpus erectus*	Ellagitannins and urolithins	*P. aeruginosa* and *Yersinia enterocolitica*	[90,91]
Roundworm	*Caenorhabditis elegans*	Exudates	LuxR	[92]
Soil-freshwater alga	*Chlamydomonas reinhardtii*	Lumichrome	*Sinorhizobium meliloti*	[93,94]
TCMs *	Rhubarb	Emodin	*P. aeruginosa* and *S. maltophilia*	[95]
TCMs	*Scutellaria baicalensis*	Flavonoid (baicalein)	TraR and RhlR	[96]
**Inhibitors against PQS System**				
Fungi	*Candida albicans*	Farnesol (sesquiterpene)	PqsA	[97]
**Inhibitors against AI-2 and AI-3 System**				
Marine alga	*Delisea pulchra*	Furanone and its derivatives	LuxR and LuxS	[98,99]
Plant	Many plants	Cinnamaldehyde and its derivatives	LuxR and AI-2	[100,101,102]
Plant	Broccoli	Quercetin	AI-2 and AI-3	[103]
Plant	Grapefruit	Limonoids (obacunone)	EHEC	[104]
**Inhibitors against AIP System**				
Bacteria	*Lactobacillus reuteri*	Cyclic dipeptides: cyclo(l-Phe-l-Pro) and cyclo(l-Tyr-l-Pro)	*agr* system	[105]
Marine bacteria	*Photobacterium*	Cyclodepsipeptides (solonomide a, b)	*agr* system	[106]
Plant: witch hazel	*Hamamelis virginiana*	2,5-di-*O*-galloyl-d hamamelose	RNAIII	[107]

* TCMs: traditional Chinese medicines.

Other marine microorganisms also produce QS inhibitors (Table 2). The Gram-positive bacterium, *Halobacillus salinus*, produces two compounds, namely *N*-(2-phenylethyl)-isobutyramide and 3-methyl-*N*-(2-phenylethyl)-butyramide, which are capable of inhibiting violacein biosynthesis of C*hromobacterium violaceum* CV026 and GFP production of *Escherichia coli* JB525 in the presence of exogenous AHLs [59]. Both Gram-positive *Bacillus cereus* and Gram-negative *Marinobacter* sp. SK-3 produce diketopiperazines (DKPs) which inhibit AHL-dependent QS [60,61]. Additionally, piericidin derived from marine actinobacteria inhibits violacein biosynthesis in *C. violaceum* CV026 [58,108].

Another source for QS inhibitors is marine algae. The red alga *Delisea pulchra* produces a number of halogenated furanones with antifouling and antimicrobial properties [98,109]. Furanones are supposed to bind AHL receptors due to the five-membered lactone scaffold structurally similar with AHLs. A series of synthetic derivatives of native furanones have been reported to interfere with AHL-mediated QS in many bacteria [99,110]. It was suggested that furanones may act to destabilize the AHL-dependent transcriptional activator LuxR of *V. fischeri* [111] and attenuate the DNA-binding activity of LuxR of *V. harveyi* [112]. Moreover, it has been found that furanones may affect the AI-2 circuit in Gram-negative and Gram-positive bacteria by covalently modifying and inactivating AI-2 synthase LuxS [113]. The protective effects of halogenated furanones in rotifers, brine shrimp and rainbow trout against pathogenic *Vibrio* species [114,115,116] and in mice against *P. aeruginosa* lung infection [117] have been demonstrated, though some furanones were found to be toxic to rainbow trout, rotifers and human fibroblasts [115,116]. Therefore, studies have focused on the synthesis of more effective and less toxic furanone derivatives that may promote their commercial or therapeutic use. Except for furanones, a mixture of floridoside, betonicine and isethionic acid, produced by the marine red alga, *Ahnfeltiopsis flabelliformes*, may also inhibit the reporter strain *A. tumefaciens* NTL4 (pCF218) (pCF372) responding to 3OC6-HSL [70]. In addition, extracts of several marine micro-algae are capable of inhibiting the QS-dependent responses of reporter strains *E. coli* JB523 and *V. harveyi* JMH612 [118].

#### 3.1.2. Terrestrial-Derived Inhibitors against AHL-Dependent QS

Various natural products from a wide range of terrestrial organisms have been demonstrated to show inhibitory activity against AHL-dependent QS. Most of these bioactive substances are derived from plants, although some originate from bacteria, fungi and insects (Table 2). Many health-benefit food sources and traditional medicines, such as fruits, herbs and medicinal plants, have received special attention as potential sources of QS inhibitory compounds. However, only a few of the compounds have been isolated or structurally and biochemically identified. A variety of phenolic compounds showed inhibitory activity against AHL-dependent QS. Among them, the flavonoids comprise a well-studied group. Flavonoids are widely produced by plants and also exist in plant-related products, e.g., propolis and honey [119]. Flavonoids display many pharmacological activities and present structural divergence [119]. The flavan-3-ol catechin, isolated from *Combretum albiflorum* bark, is the first identified flavonoid compound and is capable of reducing the production of virulence factors in *P. aeruginosa* PAO1 by interfering with RhlR [81]. Several other flavonoids from citrus and traditional Chinese medicines inhibit QS in bacteria [82,106,120]. Additionally, honey and propolis are able to interfere with QS in *C. violaceum* and *P. aeruginosa*, respectively, which may be attributed to the high abundance of flavonoids in honey and propolis [119]. Hydrolysable tannins are another group of phenolic compounds; ellagitannins or tannin-rich fraction from various plants showed QS inhibitory activity against *C. violaceum* or *P. aeruginosa* [90,91].

#### 3.1.3. Natural Inhibitors against Other QS Systems

Compared with the plentiful natural AHL-dependent QS inhibitors, natural inhibitors against other QS systems are rarely reported (Table 2). Peptide-based compounds and the phenolic compound hamamelitannin are capable of blocking *agr*-dependent QS of *Staphylococcus* spp. [105,106,107]. It is striking that farnesol (a common sesquiterpene), applied by the opportunistic pathogen *C. albicans* as a QS signal, inhibits the PQS circuit of *P. aeruginosa* by promoting non-productive interaction between PqsR and the *pqsA* promoter [97]. It was suggested that farnesol may play a role in interkingdom communications [97]. Besides farnesol, two other sesquiterpene derivatives, drimendiol from *Drimys winteri* and sesquiterpene lactones from *Centratherum punctatum* (Argentine herb), were identified as AHL-dependent QS inhibitors [83,87]. Referring to the AI-2 system, furanone, cinnamaldehyde and their derivatives may be the most effective inhibitors. Cinnamaldehyde (CA) is widely used as a flavoring substance in food chemistry [100]. Low concentrations of CA were previously found to be effective at inhibiting both AHL and AI-2 dependent QS in *V. harveyi* [101]. Subsequently, mobility shift assays revealed that CA could decrease the DNA-binding ability of LuxR of *V. harveyi*. CA and its analogs could increase significantly the survival of the nematode *Caenorhabditis elegans*, brine shrimp and giant freshwater prawn, *Macrobrachium rosenbergii*, infected with pathogenic *Vibrio* spp. [100,102,121].

#### 3.1.4. Evaluation of Natural QS Inhibitors

Over the last decade, many natural substances have been evaluated for their ability to interfere with QS. QS inhibitors isolated from natural products are excellent resources for developing potent antivirulence drugs insofar as they may provide novel scaffolds for drug design. The natural *D. pulchra* furanone compounds are unable to inhibit the QS of *P. aeruginosa*, but modified furanone analogs enhance the inhibitory effectiveness against this organism [117]. Honaucin A from the cyanobacterium *Leptolyngbya* was recently identified as an inhibitor against QS of *V. harveyi* and *V. fischeri* [57], whereas two synthesized honaucin A derivates, 4'-bromohonaucin A and 4-iodohonaucin A, showed an increased QS inhibitory activity of nearly 30-fold compared to that of honaucin A [57]. Despite these remarkable discoveries, three factors hinder the development of novel antivirulence therapies based on these bioactive substances.

Firstly, there is an increasing requirement for standardization to verify the true QS inhibitory activity. In some cases, the QS inhibitory activities of bioactive substances have been challenged because of the lack of suitable methodologies. Several QS reporter strains are widely used to identify QS inhibitors. Recently, Defoirdt *et al.* [122] detailed the inherent drawbacks of these assays. In reporter strains, the QS-regulated phenotypes are often co-dependent on other factors and/or dependent on the metabolic activity of the cells, and may thus be directly interfered with by candidate compounds. Hence, the same biosensor strain with a QS-independent expression of reporter genes should be used for adequate control experiments to verify that there is no effect imposed by candidate compounds on the particular phenotype. Additionally, QS-regulated phenotypes may be affected if the candidate compounds are toxic against reporter strains. In most cases, toxicity is only assessed by evaluating the effect on growth in a complex growth medium, which may cause false positives. For example, pyrogallol was found to be capable of inhibiting bioluminescence in a *V. harveyi* *luxN* mutant without affecting growth in a complex medium and therefore claimed as an AI-2 QS inhibitor [123]. However, it was subsequently found that pyrogallol inhibited bioluminescence when expressed constituently in an engineered strain, and the pyrogallol-related effect was abolished by the addition of catalase [124]. The apparent QS inhibitory activity of pyrogallol was demonstrated as a side effect of peroxide production [124]. Hence, more sensitive toxic assays towards bacterial cells are required [122].

Secondly, further studies on natural crude extracts with identified QS inhibitory activity are required. In many cases, the precise compounds of the bioactive molecules have not been elucidated. As mentioned above, natural inhibitors are resources for drug design to develop more potent antivirulence drugs, and purification of individual compounds is necessary to improve the likelihood of understanding the mechanisms of inhibition [39].

Moreover, the active mechanisms of these compounds are poorly understood. Successful drug design relies much on the knowledge of molecular mechanism of the connection between native signals or inhibitors and synthase or receptor, such as binding sites, conformational change and affinity change. Currently, the X-ray crystal structures of some AHL-bound LuxR-type receptors and computational protein docking methods provide powerful tools to determine the molecular mechanisms of interaction. However, the molecular mechanisms of only a few effective natural QS inhibitors have been studied in depth. The lack of information of binding interactions has thwarted the rational design of a more potent QS inhibitor.

### 3.2. Macromolecular QQ Agents

Various macromolecular agents have been found to possess the capability to quench QS. Unlike small molecular QS inhibitors, macromolecular QQ agents interfere with QS mostly through degrading signals rather than competitively binding to signal receptors. Most of the identified macromolecular QQ agents target the AHL-dependent QS, although enzymatic degradations of DSF, PQS and AI-2 have also been reported. *Bacillus*, *Staphylococcus* and *Pseudomonas* possess DSF inactivation activity [125]. The 2,4-dioxygenase, Hod, involved in quinaldine utilization pathway in *Arthrobacter nitroguajacolicus* is able to cleave PQS [126]. In the Lsr-type AI-2 system, cytoplasmic enzyme LsrK is responsible for phosphorylation of AI-2 and phospho-AI-2 is unstable [127]. When LsrK is artificially provided *in vitro*, the extracellular phosphor-AI-2 molecules cannot be transported into cells and are degraded overnight. LsrK-mediated degradation of AI-2 attenuates the QS response in *S. enterica* serovar Typhimurium and *V. harveyi* [127].

To date, many macromolecular QQ agents against AHL-dependent QS have been reported. Generally QQ enzymes are a major portion of the macromolecular agents, although a few antibodies have been generated to interfere with AHL-dependent QS through sequestration or hydrolyzation of AHLs [39]. Enzymatic degradation of AHLs has been extensively studied, and found in many organisms including mammals, plants, fungi, achaea and bacteria [39,128], although the genes responsible for AHL-degrading activity in plants and fungi have not been identified. A comprehensive summary of AHL-degrading bacteria with marine or terrestrial origin was provided in our previous publication [129].

AHL-degrading enzymes may be classified into three major types according to their enzymatic mechanisms: AHL lactonase (lactone hydrolysis), AHL acylase (amidohydrolysis) and AHL oxidase and reductase (oxidoreduction). AHL lactonase hydrolyzes the ester bond of AHL yielding the corresponding *N*-acyl-homoserine. This hydrolyzation may also occur spontaneously at alkaline pH, and may be reversed under acid pH [40]. AHL acylase hydrolyzes the amide bond of AHL to yield a homoserine lactone and the corresponding fatty acid chain, whereas AHL oxidase and reductase usually catalyzes a modification of AHLs. In most cases, AHL lactonases require metal ions (except AiiM and QsdH) and target both short and long acyl chain AHLs. Unlike lactonases, acylases exhibit substrate specificity based on the length of the acyl chain and the substitution on the β position of the AHL chain.

#### 3.2.1. AHL Lactonases

**Figure 3:**
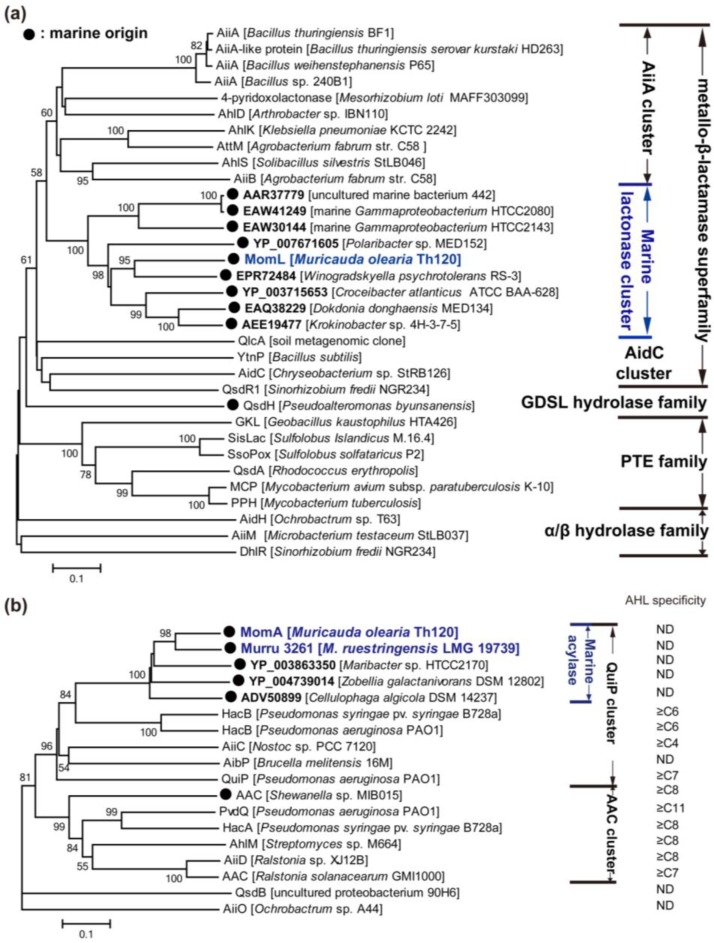
Neighbour-joining tree of *N*-acylhomoserine lactone (AHL) enzymes based on amino acid sequences. Each of these AHL lactonases was experimentally identified, except the members named with accession number in Genbank (bold). MomL, MomA and Murru 3261 were identified by us recently (blue colored). The dendrogram was constructed by neighbor-joining method with the MUSCLE program in the MEGA software package (1000 bootstrap replicates). Bootstrap coefficients below 50% were not shown. Scale bar, 0.1 substitutions per amino acid position. Marine clusters were colored in blue. (**a**) Tree of AHL lactonase; (**b**) tree of acylase. ND: not determined.

To date, approximately 30 types of AHL lactonases have been identified (except for predicted AHL lactonases or highly similar enzymes in one genus). According to the amino acid sequences, these lactonases belong to the metallo-β-lactamase superfamily, the phosphotriesterase (PTE) family and other particular families (Figure 3a). Among them, the AHL lactonases of the metallo-β-lactamase superfamily have been most extensively studied, and are widespread in various bacterial species. The metallo-β-lactamase group may be further classified into several clusters, *i.e.*, AiiA, AidC and a novel marine AHL lactonase cluster [130] (discussed in depth later on) according to the phylogenetic tree (Figure 3a).

The first identified AHL lactonase AiiA (autoinducer inactivation) from *Bacillus* sp. 240B1 belongs to the metallo-β-lactamase family. AiiA was initially thought to hydrolyze the amide linkage between homoserine lactone and acyl side chain because metallo-β-lactamase can cleave the amide bond of the penicillin β-lactam ring. However, it was demonstrated later that AiiA degraded the ester rather than amide bond [131]. The *aiia* homogenous genes are widespread in *Bacillus* species. The molecular mechanism of AHL degradation has been revealed. AiiA amino acid sequence contains a “^104^HXHXDH^109^~60aa~H^169^” motif, which is common in metallo-β-lactamases. Single-residue mutagenesis has shown that His 106, Asp 108, His 109 and His 169 are necessary for AiiA activity [131]. AiiA is a metalloprotein binding two equivalents of zinc, which is necessary for its activity [132]. Crystal studies of AiiA from *B. thuringiensis* showed the dinuclear zinc binding sites of AiiA: Zn 1 binds to His 104, His 106 and His 169, whereas Zn 2 binds to Asp 108, His 109 and His 235 [133]. These two Zn^2+^ ions, which are separated by a distance of 3.4 Å, are bridged by a hydroxide ion and the Oδ2 atom of Asp191 [133]. Additionally, Tyr 194 is essential for activity, and may be able to stabilize the substrate’s carbonyl and/or a tetrahedral intermediate formed subsequent to hydroxide attack [133,134]. AiiA shows a broad substrate specificity and a preference for substrates with long acyl chain AHLs [135], however, C6-HSL is mostly used as substrate in crystal studies of AiiA [133,134]. Recently, another crystal study of a F107W mutation of *B. thuringiensis* AiiA revealed an unexpected interaction with the ring-opened product. Two aromatic residues, Phe 64 and Phe 68, form a hydrophobic clamp, centered around the seventh carbon of the decanoyl chain of ring-opened C10-HSL, making an interaction that would be available for longer substrates but not for shorter ones [136]. Although zinc was found in native AiiA, dicobalt-, dimanganese- or dicadmium-substituted AiiA exhibits hyperactivity compared with that of dizinc-substituted enzyme [134,137]. Heterologous expression of *aiia* in numerous pathogenic bacteria, including *P. aeruginosa*, *B. thailandensis* and *E. carotovora*, may reduce AHL accumulation and decrease their virulence expression, which indicates the potential use of AiiA as a strategy for antivirulence therapy [131].

Two members of AiiA cluster, AttM and AiiB, showing 32% and 28% identity to AiiA_240B1_, were found in the plant pathogen *A. tumefaciens* C58, and their corresponding coding genes *attm* and *aiib* (an *attm*-paralogous gene) located on the pAt plasmid and the pTi plasmid [138], respectively. Both AttM and AiiB have the “HXHXDH” motif and AiiB appears to be a metal-dependent AHL lactonase with broad substrate specificity [139]. *A. tumefaciens* infects a broad range of dicotyledonous plants and may transfer an oncogenic DNA fragment, the T-DNA, from its tumor-inducing plasmid to the nuclear genome of the plant hosts. These processes are regulated by TraI/R QS circuit, lactonases AttM and AiiB [140,141]. During the early process of *A. tumefaciens* infection in wounded plant hosts, the expression of *attm* is promoted by γ-aminobutyric acid, a molecule synthesized for defense by plants, and thereby the 3OC8-HSL-dependent virulence expression is attenuated. However, in the growing tumor, the high synthesis rate of 3OC8-HSL and the moderated expression of *attm* caused by plant produced l-proline permit the expression of QS-regulated functions including the transfer of Ti plasmid by conjugation [141].

The AidC cluster contains AidC and QsdR1 encoded by *Chryseobacterium* sp. StRB126 and *Sinorhizobium fredii* NGR234, respectively, and additionally includes YtnP, another AHL lactonase from *Bacillus* species [142,143,144]. Interestingly, the expression of *ytnP* in *B. subtilis* may be induced by streptomycin, and YtnP may conversely inhibit the production of streptomycin and the development of aerial mycelium in *Streptomyces griseus. B. subtilis* may activate *ytnP* expression in response to the presence of certain antimicrobial compounds as a defensive strategy against threatening bacteria since it would allow *B. subtilis* to selectively inhibit the QS-regulated behaviors of harmful microbial communities [144]. Furthermore, most of the AHL lactonases (both in the AiiA cluster and the AidC cluster) belonging to metallo-β-lactamase family are soil-derived. In this connection, we recently identified many marine bacteria with AHL-degrading activity [129], and one of these organisms, *Muricauda olearia* Th120, possesses a gene encoding a novel AHL lactonase which represents a new cluster of AHL lactonase in the metallo-β-lactamase family [130] (Figure 3a).

Apart from members of the metallo-β-lactamase family, another group of AHL lactonases shares key sequence and active site features with phosphotriesterases (PTEs), and these lactonases are termed PTE-like lactonases (PLLs). PLLs exist in both bacteria and archaea. Afriat *et al.* [145] discovered three PLLs, *M. tuberculosis* PPH, *R. erythropolis* AhlA and *Sulfolobus solfataricus* SsoPox. Although no significant sequence identities are shared with AiiA, PLLs show a wide range of AHL-degrading capability and require metal ions for their activities [145]. Crystal studies have revealed that the hyperthermophilic SsoPox shows a high level of similarity with the structure of AiiA [146]. Significantly, the production of AHLs and virulence factors of *P. aeruginosa* PAO1 may be greatly reduced in the presence of SsoPox-immobilized membranes [147]. The thermostability of SsoPox [147], as well as GKL of *Geobacillus kaustophilus* [148] and SisLac of *S. islandicus* [149], is an advantage for their biomedical applications. However, most PLLs showed relatively lower AHL-degrading activities than AiiA [147,148,149]. Curiously, all the identified PLLs exhibit promiscuous phosphotriesterase activities [147,148,149]. Bacterial PTEs belong to the amidohydrolase superfamily, a highly diverse superfamily with many different hydrolytic activities. They harbor a degrading rate approaching the diffusion limit (*k*_cat_/*K*_M_ ≥ 4 × 10^7^) for their best substrate paraoxon, which is a widely used pesticide introduced in the 20th century. PTEs could have therefore evolved from a member of the PLL family, utilizing its latent promiscuous paraoxonase activity as an essential starting point [145]. Additionally, DhlR, AidH and AiiM can be temporarily sorted into one group because they belong to the alpha/beta hydrolase family (Figure 3a). Both AidH and AiiM are capable of degrading short- and long-chain AHLs in an unknown and metal-independent mechanism [150,151]. There is another non-bacteria-derived group of AHL lactonases, paraoxonases 1, 2, and 3, which are prevalent in mammalian cells [128]. They all catalyze lactone hydrolysis, but differ in their substrate specificity [128].

#### 3.2.2. AHL Acylases

AHL acylases have been found in bacteria including *Pseudomonas*, *Ochrobactrum*, *Arthrobacter*, *Streptomyces*, *Nostoc* and *Brucella* (Figure 3b). The AiiD of *Ralstonia* sp. XJ12B isolated from a biofilm in an experimental water treatment system is the first identified AHL acylase [152], although an AHL acylase-like activity was previously detected in *Variovorax paradoxus* VAI-C which degrades and utilizes multiple AHLs as the sole source of carbon, nitrogen and energy [153]. AiiD belongs to the Ntn (*N*-terminal nucleophile) hydrolase superfamily, and shares 22%–24% identities with several cephalosporin and penicillin acylases [152]. Actually, AiiD degrades several AHLs, rather than penicillin G or ampicillin, indicating that AHLs are its unique substrates. In comparison, AhlM from *Streptomyces* sp. which shows 35% identity with AiiD in the deduced amino acid sequence, was capable of degrading penicillin G and long-chain AHLs [154]. The gene responsible for AHL-acylase activity in *V. paradoxus* was not identified until whole genomic sequencing of *V. paradoxus* revealed recently a putative AHL acylase gene (Vapar_3883 of *V. paradoxus* S110) [155]. However, biochemical studies are required to confirm its activity. Like AHL lactonase, AHL acylase is considered to have the potential to interfere with QS of bacterial pathogens. Expression of *aiiD* in *P. aeruginosa* PAO1 weakened its ability to swarm, to produce elastase and pyocyanin, and to paralyse nematodes [152]. The addition of AhlM to the growth medium for *P. aeruginosa* also reduced the accumulation of AHLs, and decreased the production of virulence factors including elastase, total protease and LasA [154].

*P. aeruginosa* PAO1 was previously found to utilize AHLs for growth, so far three proteins (PvdQ, QuiP, and HacB) belonging to the Ntn hydrolase have been characterized biochemically [156]. Among them, PvdQ (PA2385) is the most extensively studied AHL acylase. PvdQ is expressed as a proenzyme that is auto-proteolytically activated by post-translational cleavage resulting in the excision of a 23-residue prosegment and the formation of an 18 kDa α-chain and a 60 kDa β-chain [156]. The mature PvdQ can hydrolyze the amide bond of AHLs, and demonstrates substrate specificity for long-chain AHLs [156]. However, it is the gene *quiP* (*pa1032*) rather than *pvdQ* that is responsible for the ability of *P. aeruginosa* PAO1 to utilize AHLs as a sole carbon and energy source for growth [156,157]. Additionally, another AHL acylase HacB (PA0305) can degrade AHLs with acyl chains ranging in length from 6 to 14 carbons [158]. The physiological function of each of the three acylases in *P. aeruginosa* PAO1 is intriguing. PvdQ was found to be expressed only when iron is present at very low concentrations [159]. Mutation of *pvdQ* did not affect the growth of *P. aeruginosa* but abrogated pyoverdine production and greatly affected swarming motility and biofilm formation at low iron concentrations [159]. Moreover, the virulence of *pvdQ* mutant against *C. elegans* was reduced. All of these data indicate that PvdQ plays an essential role in siderophore biosynthesis, on which *P. aeruginosa* depends for growth in iron-limited environments [159]. Therefore, PvdQ is a target for antivirulence therapy and different synthetic inhibitors are able to block its activity [160]. Nevertheless, the physiological functions of QuiP and HacB are still unknown.

To date, most of the identified AHL acylases belong to the Ntn hydrolase superfamily, except QsdB and AiiO which belong to the amidase family and α/β hydrolase fold family, respectively [161,162]. AHL acylases belonging to the Ntn hydrolase superfamily may also be classified into two clusters according to the phylogenetic tree (Figure 3c). These are referred to as AAC and QuiP clusters, respectively. The substrate specificity of each acylase cluster was also summarized, and it was speculated that the QuiP cluster might degrade a broader range of AHLs than the AAC cluster because some members of the QuiP cluster degrades C6-HSL even C4-HSL whereas those of AAC cluster could only degrade AHLs longer than C8-HSL. Additionally, most of these AHL acylases are located in the periplasmic space, whereas AhlM and HacB are secretory.

#### 3.2.3. AHL Oxidoreductases

Compared to the abundant data of AHL lactonases and acylases, there are fewer reports about inactivation of AHLs by the modification of chemical structure of AHLs; only a few AHL oxidoreductases have been identified thus far. Because signal receptors usually respond to specific AHLs, the modification might affect the signal recognition, and thereby interfere with QS-regulated functions. Bacteria-derived AHL reduction activity was first discovered in *Rhodococcus erythropolis* in which AHLs with 3-oxo substituents were rapidly degraded by reduction of the keto group at the β position, yielding the corresponding 3-hydroxy derivative AHLs [163]. However, the gene responsible for this activity has not yet been identified. CYP102A1 from *Bacillus megaterium*, a widely studied cytochrome P450, is the second identified AHL oxidoreductases that oxidizes AHL at the ω-1, ω-2, and ω-3 carbons of the acyl chain [164]. Furthermore, this oxidation activity is very efficient towards ring-opened AHLs and fatty acid chains which are the corresponding products of AHL lactonase and acylase, respectively [164]. The third oxidoreductase, the NADH-dependent enzyme BpiB09, was identified by metagenomic analysis. Expression of *bpiB09* in *P. aeruginosa* reduced its swimming motilities, pyocyanin production, biofilm formation and thereby the pathogenicity to *C. elegans* [165]. Moreover, *Burkholderia* sp. GG4, isolated from ginger rhizosphere, was previously found to possess a unique AHL-modifying activity that reduces 3-oxo-AHLs to 3-hydroxy-AHLs [166] although the responsible gene has not been identified. The complete genome of this strain reported recently might reveal its responsible gene [167]. AHL can also be enzymatically inactivated by haloperoxidases from *D. pulchra* [168], *Laminaria digitata* [169] and *Nitzschia cf pellucida* [170] via a H_2_O_2_-dependent mechanism.

## 4. Microorganisms May Produce QQ Agents to Gain Benefits in a Competitive Environment

Microorganisms exist in a multi-species and competitive environment, and have developed many survival strategies to gain benefits and compete for space, nutrition and ecological niches. QS is possibly one critical strategy used for competition by microorganisms to synchronize and coordinate social behaviors. Many of these behaviors (e.g., the production of antimicrobial compounds) are primarily advantageous when expressed by a group of bacteria but seemingly futile if performed by a single bacterium [171], though QS-regulated processes can also be induced in single cells in a confined environment [10,11]. Whereas, for other microorganisms exposed to QS-regulated competitive determinants, the selective pressure may drive the evolution of defensive mechanisms of fighting with competing species. It may be assumed that one microbial species may evolve two possible strategies to fight with another species that produces an antimicrobial compound in a QS-regulated mechanism. One strategy is to develop antimicrobial compound-degrading enzymes or other antimicrobial compound-resistant mechanisms. Another conceivable strategy is to interrupt the QS of competing species [171]. The hypothesis of QS interruption is straightforward because QQ-agent-producing bacteria can inhibit the QS-regulated behaviors of competing species and therefore gain benefits or avoid being killed. In this situation, small molecular QS inhibitors should be secreted outside of cells since their targets (e.g., signal synthases or receptors) are located in the membranes or cytoplasm of competing cells, whereas QQ enzymes could be either secreted or cytoplasmic because the signal molecules are diffusible. However, the cellular localization of QQ compounds has been little investigated.

The discovery of co-existence of QS and QQ bacteria in various environments might provide supports for this hypothesis [166,172,173]. However, conclusive empirical evidence has not been demonstrated regarding the relationship between QQ agents and the benefits gained in the natural environment. Additionally, some laboratory co-cultures of QS and QQ bacteria may provide evidence. The pyocyanin, which is a QS-regulated product of *P. aeruginosa*, is toxic to *S*. *delphini* [174] that produces two AHL-dependent QS inhibitors (yayurea A and B) and protects itself from killing by *P. aeruginosa* via suppressing the production of pyocyanin [72]. Likewise, pyocyanin is toxic to *C. albicans* [175]. With the production of farnesol, *C. albicans* blocks the PQS circuit and thus the pyocyanin biosynthesis of *P. aeruginosa* [97]. Farnesol-like molecules are ubiquitous in the natural environment, and are able to interrupt the PQS circuit, which suggests that other organisms may have the potential to moderate *P. aeruginosa* virulence [97]. It seems likely that the benefits outweigh the costs for these microorganisms to produce compounds to prevent harmful QS-regulated activities of other bacteria and thereby gain space and other resources within microbial communities.

The interference of QS exists not only between different species but also between different strains of the same species.In Gram-negative bacteria, the native AHL utilized by *C. violaceum* ATCC 31532 is C6-HSL but the QS-regulated violacein production can be inhibited by long-chain AHLs produced by *C. violaceum* ATCC 12472 or other bacteria [176,177]. Gram-positive *S. aureus* utilizes four different groups of AIPs. Each can specifically activate its cognate AgrC receptor, but inhibit all others by competitive binding to the non-cognate receptors [178,179,180,181]. This form of QS inhibition was suggested as a strategy for microorganisms to occupy specific niches during infection.

The physiological function of QQ enzyme has been discussed repeatedly but is still unclear [182,183,184]. PvdQ of *P. aeruginosa* participates in siderophore biosynthesis [159]. The major role of AttM in *A. tumefaciens* is for the degradation of γ-butyrolactone rather than regulation of AHL accumulation, and the AHL-degrading activity might be only a side effect [185]. *V. paradoxus* and *Arthrobacter* sp. utilize AHLs as a source of nitrogen or/and carbon for growth depending on their AHL-degrading enzymes [153,186]. AiiA is essential for rhizosphere colonization of *B. thuringensis* [187]. However, these results are insufficient to explain the physiological functions of QQ enzymes in these QQ bacteria that do not harbor the AHL-dependent QS and cannot utilize AHL to grow. Recently, Schneider *et al.* [144] reported that the expression of YtnP in *B. subtilis* was induced by streptomycin, an antibiotic produced by the *Streptomyces* species. Conversely, YtnP inhibits the production of streptomycin in *S*. *griseus* probably by degrading its QS signaling molecule γ-butyrolactone [144]*.* The streptomycin-induced expression of YtnP may allow *B. subtilis* to response to certain antimicrobial compounds and selectively inhibit the QS of harmful microorganisms before being killed. It seems likely that QQ enzyme could allow its producers to obtain competitive advantages over competitors in natural ecosystems.

The hypothesis that microorganisms produce QQ agents in order to gain competitive advantage is not sufficient to explain all of these discoveries. Although several examples showed that the QQ agent producers could survive or even gain benefit through inhibiting QS-regulated harmful behaviors of their competitors, the possibility of “accidental” QQ activity of some QQ agents still exists.

## 5. QQ in the Marine Environment: A Tremendous Resource to Be Developed

QQ may be a strategy used by microorganisms to gain benefit in a competitive environment. Also, it is believed that in the highly diverse marine ecosystem, microorganisms with capabilities of producing small QS inhibitors and QQ enzymes remain to be discovered. Romero *et al.* [184] proposed that QQ is likely to be a common activity in marine bacteria because a high abundance of QQ bacteria was found among marine cultivable bacteria [188] and a high frequency of QQ genes was discovered in marine metagenomes. In our previous study, 25 marine QQ strains belonging to 14 bacterial species were obtained and it is noteworthy that the QQ activities in 12 species had not been reported previously [129]. Although only a few studies have been carried out to assay the AHL-degrading activity of marine bacteria, more than 30 species of QQ bacteria belonging to *Alphaproteobacteria*, *Gammaproteobacteria Actinobacteria*, *Flavobacteriia* and *Firmicutes* have been identified thus far (Table 3). Additionally, some QQ strains have revealed degradative activity only against long-chain AHLs. Because AHL lactonases normally present broad AHL inactivating activities while many acylases are specific to long-chain AHLs, we assumed that AHL acylases might be more common than lactonases in the ocean. This is consistent with the distribution of acylase and lactonase coding sequences in metagenome collections [184]. Therefore, many marine QQ bacteria may be still undiscovered, and the prevalence of QQ enzymes in marine bacteria may be higher than expected.

The high diversity and abundance of marine QQ bacteria may lead to the discovery of new QQ enzymes and AHL-degrading mechanisms. However, few responsible genes in these bacteria have been identified (Table 3). One of our identified QQ bacteria, *M. olearia* Th120, showed strong AHL-degrading activity, and further studies revealed a novel AHL lactonase and a novel AHL acylase [130] (Figure 3). The identities of the amino acid sequence of AHL lactonase MomL (*Muricauda olearia* marine AHL lactonase) to known lactonases are below 30%, and the top eight strains (with identity higher than 39%) using BLASTP against the NR protein database are also typical marine bacteria. Therefore, MomL was believed to represent a new class of AHL lactonase, which may be widespread in the marine environment. Likewise, AHL acylase MomA (*Muricauda olearia* marine AHL acylase) may represent a marine-derived AHL acylase. It is even more astounding that the ethyl acetate extracts of Th120 culture showed inhibitory activity in *A. tumefaciens* A136 plate assay [130]. All of these findings indicate that marine microorganisms may be important resources for the discovery of new antivirulence strategies. Therefore, an increasing effort is needed in the discovery of new natural QQ agents from marine microorganisms.

**Table 3:** Marine quorum quenching bacteria.

Strain	AHL-Degrading Ability *	Activity **	Origin	Reference
*Actinobacteria*				
*Rhodococcus erythropolis* strains	C4, C6, C10 and 3OC12	Lactonase	*Fucus vesiculosus* and sediment	[188]
*Alphaproteobacteria*				
*Hyphomonas* sp. USC2	C4, C6, C10 and 3OC12	Lactonase	*Fucus vesiculosus*	[188]
*Marivita* sp. Th30	C6, C12 and C14	ND	Flounder	[129]
*Novosphingobium* sp. Th20	C6-C14 and 3OC6-3OC14	ND	Flounder	[129]
*Paracoccus* sp. PP2-663	C4-C12	ND	Manila clam	[189]
*Phaeobacter* sp. USC177	C4, C6, C10 and 3OC12	ND	*Fucus vesiculosus*	[188]
*Rhodobacter* sp. Th15	C8-C14 and 3OC14	ND	Flounder	[129]
*Roseovarius* aestuarii USC61	C4, C6, C10 and 3OC12	Lactonase	Water tank	[188]
*Sphingopyxis flavimaris* T51	C6-C14 and 3OC10-3OC14	ND	Flounder	[129]
*Sphingopyxis litoris* th8	C6-C14 and 3OC6-3OC14	ND	Flounder	[129]
*Stappia* sp. USC176	C4, C6, C10 and 3OC12	Lactonase	*Fucus vesiculosus*	[188]
*Stappia* sp. USC5	C4, C6, C10 and 3OC12	Lactonase	*Fucus vesiculosus*	[188]
*Firmicutes*				
*Bacillus circulans* USC24	C4, C6, C10 and 3OC12	Lactonase	Sediment	[188]
*Bacillus* sp. KT7	C6-C14, 3OC8-3OC12 and 3OHC8-3OHC12	ND	Intertidal rocks colonized by *Ulva*	[190]
*Bacillus* spp.	C6	ND	Shrimp and bass	[191]
*Oceanobacillus* spp.	C4, C6, C10 and 3OC12	Lactonase	*Fucus vesiculosus*	[188]
*Flavobacteria*				
*Flaviramulus ichthyoenteri* Th78	C6-C14 and 3OC6-3OC14	Lactonase	Flounder	[129]
*Maribacter* sp. 139	C4, C6, C10 and 3OC12	Lactonase	Ocean water	[184]
*Muricauda olearia* Th120	C6-C14 and 3OC6-3OC14	Latonase and acylase	Flounder	[129]
*Olleya marilimosa* 138E	C4, C6, C10 and 3OC12	Lactonase	Ocean water	[184]
*O. marilimosa* t168	C6-C14 and 3OC6-3OC14	Lactonase	Marine	[129]
*Tenacibaculum discolor* 20J	C4, C6, C10 and 3OC12	Lactonase	Sediment	[188]
*T. discolor* t84	C6-C14 and 3OC6-3OC14	ND	Gill of flounder	[129]
*T. maritimum* 2154^t^	C10	Acylase	Fish farm disease	[192]
*T. soleae* strains	C6-C14 and 3OC6-3OC14	Lactonase	Gill of flounder	[129]
*Gammaproteobacteria*				
*Alteromonas marina* PP2-67	C4-C12	ND	Pod razor clam	[189]
*Alteromonas* sp. USC168	C4, C6, C10 and 3OC12	ND	*Fucus vesiculosus*	[188]
*A. stellipolaris* pp2-644	C4-C12	ND	Carpet-shell clam	[189]
*Colwellia aestuarii* T171	C8-C14 and 3OC10-3OC14	ND	Gill of flounder	[129]
*Glaciecola* sp. B20	C10-C14, 3OC10-, 3OHC10, 3OC12 and 3OHC12	ND	Intertidal rocks	[190]
*Halomonas taeanensis* USC33	C4, C6, C10 and 3OC12	Lactonase	Sediment	[188]
*Marinobacterium* sp. B2	3OC10, C12, 3OC12, 3OHC12 and C14	ND	Intertidal rocks	[190]
*Pseudoalteromonas byunsanensis* 1A01261	C4-C14 and 3OC4-3OC12	Lactonase	Marine	[193]
*P. rydzensis* Th125	C10-C14 and 3OC10-3OC14	ND	Flounder	[129]
*Salinimonas* sp. T194	C8-C14 and 3OC10-3OC14	ND	Gill of flounder	[129]
*Salinicola salarius* 131	C4, C6, C10 and 3OC12	Lactonase	Ocean water	[184]
*Shewanella* sp. B21	C8-C14, 3OC8-3OC12 and 3OHC8-3OHC12	ND	Intertidal rocks	[190]
*Thalassomonas* sp. PP2-459	C4-C12	ND	Carpet-shell clam	[189]
*Thalassomonas* sp. T202	C8-C14 and 3OC10-3OC14	ND	Gill of flounder	[129]

* All of AHLs contain even number of carbons; ** AHL-degrading activities were identified in bacterial cultures but not purified enzymes, except for *P. byunsanensis* 1A01261 and *M. olearia* Th120; ND: not determined.

## 6. Further Issues of Concern for the Application of QQ Agents

The antivirulence activities of small molecular QS inhibitors and QQ enzymes have been demonstrated *in vitro* and *in vivo*. However, both have advantages and drawbacks due to their entirely distinct molecular structures and functional mechanisms.

QS inhibitors may target one specific signal receptor or some homogenous receptors, such as the LuxR-like family. For example, compounds 4606–4327, CTL, CL and mBTL are synthetic AHL analogs with similar structures [194]. Each is the antagonist of CviR of *C. violaceum* and LuxN of *V. harveyi*. However, only mBTL is capable of inhibiting the pyocyanin production of *P. aeruginosa* PA14 whereas CL and CTL show non-inhibitory activity [195]. This may contribute to developing drugs that are capable of preventing virulence expression in specific pathogens. However, microbial infection is often caused by multiple pathogenic species, and one drug may be insufficient in this situation. In contrast, QQ enzymes, especially AHL lactonase, are capable of degrading a wide range of AHLs, and are likely to be more efficient for antivirulence by treating multi-microbial infection. Nevertheless, this capability of AHL-degrading enzymes could cause unintended consequences if a beneficial activity of a probiotic in the intestine of animals or human is positively regulated by its AHL-dependent QS [196].

Compared with QQ enzymes, the structures of QS inhibitors are relatively simple and can be easily modified through synthetic methods. Moreover, small QS inhibitors allow for temporal control of a biological system, and this control is often rapid, depending on the diffusibility of compounds. For example, the low molecular weight of QS inhibitors facilitates their absorption by animals; they may be administered orally or intravenously like other drugs. Furthermore, the nonproteinaceous nature as well as low molecular weight of QS inhibitors can effectively prevent an antibody-based immune response unlike that of QQ enzymes.

Stability is another important issue that should be taken into account for both of these two agents. QQ enzymes could be easily proteolyzed and most of them are sensitive to heat. Likewise, QS inhibitors may be degraded by abiotic or biotic elements. This is of concern especially for some QS inhibitors possessing similar structures with native AHLs. This type of QS inhibitor is suspected to be degraded by AHL-degrading enzymes because AiiA can degrade AHL analogs [135,137], and SisLac harbors AHL lactonase activity, esterase activity and phosphotriesterase activity [149]. Many of the identified QS inhibitors have similar structures to AHLs, especially of the synthetic AHL analogs. If this type of QS inhibitor was used to inhibit QS-dependent virulence in a multi-microbial community, it may be degraded by QQ enzymes produced by other microorganisms to obtain a competitive benefit in the environment. However, the QQ enzyme-mediated degradation of QS inhibitors has been ignored to date. It is advised that whether QS inhibitors can be degraded by QQ enzymes should be determined in the criteria proposed by Defoirdt *et al.* [122] for the scientific evaluation of QS inhibitory activity.

The emergence of resistance to QQ compounds has raised doubts about whether QS is an ideal target for antivirulence therapy. Defoirdt *et al.* [197] proposed that bacteria might evolve resistance to QQ compounds because QS disruption could indeed affect bacterial growth under certain conditions (e.g., during infection of a host). Subsequently, growth inhibition was observed by cultivating *P. aeruginosa* PA14 on minimal medium using adenosine as the sole carbon source and with the simultaneous exposure to the synthetic QS inhibitor furanone C-30 [198]. Further studies revealed a QQ resistance mechanism through an increased efflux of C-30 from the cells by mutations in the *mexR* and *nalC* genes, both of which encode negative regulators of the MexAB-OprM multidrug resistance efflux pump [198]. Moreover, *mexR* and *nalC* mutations were found in several clinical *P. aeruginosa* isolates. However, unlike QS inhibitors, application of QQ lactonase would be less likely to induce QQ resistance [197,199]. The degradation activity of QQ enzyme targeting signals is extracellular rather than entering cells and targeting receptors, which would be hardly influenced by an increased efflux of compounds from cells. Despite the possible ways in which bacteria develop resistance to AHL-degrading enzymes, such as increasing production of autoinducers, synthesis of modified autoinducers and evolution of mutations with higher-affinity receptors [199], the high AHL-degrading activity and broad range of substrate specificity of QQ enzymes would reduce the possibility of evolving QQ resistance. 

The potential for the development of antivirulence drugs has been emphasized for years, and a number of QQ agents have been discovered or synthesized, but none have been marketed. Certainly, the lack of appropriate delivery systems is one challenge. The corresponding QQ agents should be introduced into the hosts against different pathogens by controlling the rate, time, and place of release. The novel biological nanofactories engineered by Fernandes and colleagues would provide a promising specific delivery of QQ agents [200]. Another self-regulating system designed to release QQ agents dependent on the titer of bacteria surrounding medical devices has potential [201]. It is likely to be more difficult to develop formulations for delivering macromolecular QQ agents due to the extremely low bioavailability of protein drugs [202]. Although oral administration of AiiA homogenous protein and preparation dry powder of PvdQ have been attempted, there are concerns about the stability of QQ enzymes. Clatworthy *et al.* [1] argued that the greatest challenge for commercialization of antivirulence drugs is not technological but economic. Since antivirulence drugs are narrow-spectrum, their effectiveness is dependent on the precise diagnosis of the pathogens to achieve an appropriate choice of compounds. Therefore, appropriate tools need to be developed to allow decisions to be made [1]. Thus, the technological and economic obstacles for the commercialization of QQ drugs to be overcome in the future become more urgent than the discovery of novel QQ agents [1].

## 7. Concluding Remarks

In summary, the utilization of quorum quenching as a promising strategy of antivirulence therapy has been demonstrated *in vitro* and *in vivo*. The natural QQ agents, especially those derived from marine microorganisms, are great resources for developing antivirulence therapy. Recently, several studies have revealed a wide spread of QQ activities in marine microorganisms, however, these QQ resources need to be explored more deeply. Therefore, further research on QQ resources and mechanisms would provide more alternatives for developing antivirulence therapy.

## Abbreviations

AHL: *N*-Acyl-homoserine lactonse
AI-2: Autoinducter-2
AI-3: Autoinducter-3
AIP: Autoinducing peptides
DSF: Diffusible signal factor
BDSF: *Burkholderia cenocepacia* diffusible signal factor
CAI-1: *Cholerae* autoinducer-1
Ea-C8-CAI-1: (*Z*)-3-Aminoundec-2-en-4-one
PQS: *Pseudomonas* quinolone signal
IQS: Integrating QS signal
*R*-THMF: (*2R*,*4S*)-2-Methyl-2,3,3,4-tetrahydroxytetrahydrofuran
*S*-THMF-borate: (*2S*,*4S*)-2-Methyl-2,3,3,4-tetrahydroxytetrahydrofuran-borate
DPD: 4,5-Dihydroxy-2,3-pentanedione
